# Alternative splicing during *Arabidopsis* flower development results in constitutive and stage-regulated isoforms

**DOI:** 10.3389/fgene.2014.00025

**Published:** 2014-02-12

**Authors:** Haifeng Wang, Chenjiang You, Fang Chang, Yingxiang Wang, Lei Wang, Ji Qi, Hong Ma

**Affiliations:** ^1^State Key Laboratory of Genetic Engineering and Institute of Plant Biology, Collaborative Innovation Center for Genetics and Development, School of Life Sciences, Fudan UniversityShanghai, China; ^2^Institutes of Biomedical Sciences, Fudan UniversityShanghai, China

**Keywords:** alternative splicing, floral development, RNA-Seq, stage transition, novel transcribed regions

## Abstract

Alternative splicing (AS) is a process in eukaryotic gene expression, in which the primary transcript of a multi-exon gene is spliced into two or more different mature transcripts, thereby increasing proteome diversity. AS is often regulated differentially between different tissues or developmental stages. Recent studies suggested that up to 60% of intron-containing genes in *Arabidopsis thaliana* undergo AS. Yet little is known about this complicated and important process during floral development. To investigate the preferential expression of different isoforms of individual alternatively spliced genes, we used high throughput RNA-Seq technology to explore the transcriptomes of three floral development stages of *Arabidopsis thaliana* and obtained information of various AS events. We identified approximately 24,000 genes that were expressed at one or more of these stages, and found that nearly 25% of multi-exon genes had two or more spliced variants. This is less frequent than the previously reported 40–60% for multiple organs and stages of *A. thaliana*, indicating that many genes expressed in floral development function with a single predominant isoform. On the other hand, 1716 isoforms were differentially expressed between the three stages, suggesting that AS might still play important roles in stage transition during floral development. Moreover, 337 novel transcribed regions were identified and most of them have a single exon. Taken together, our analyses provide a comprehensive survey of AS in floral development and facilitate further genomic and genetic studies.

## Introduction

Alternative splicing (AS) produces multiple mRNAs from pre-mRNAs with different splicing patterns, and has been detected widely in multicellular eukaryotes (Pan et al., 2008; Wang et al., 2008; Graveley et al., 2010). More than 95% multi-exon genes have been estimated to have AS variants in human (Pan et al., 2008; Wang et al., 2008). AS plays important roles in regulating gene expression and increasing transcriptome diversity and proteome complexity. AS can produce transcripts that contain premature termination codons (PTCs), which are recognized by the non-sense mediated decay (NMD). NMD causes the degradation of such transcripts, thus reducing the abundance of splice variants. In the model plant *Arabidopsis thaliana*, approximately 50% of AS events are reported to produce transcripts with PTCs; for example, isoforms of the *AFC2* and *SOC1* genes have been found to contain PTCs (Filichkin et al., 2010; Marquez et al., 2012).

A number of studies suggested that AS can affect important biological processes in plants, including biotic/abiotic stress responses, photosynthesis, defense responses (Reddy, 2007), metabolic pathways (Gorlach et al., 1995), catabolic pathways (Kopriva et al., 1995), and flowering. Quesada et al. found that AS of *FCA* pre-mRNA prevented precocious flowering, by reducing its expression via cleavage and polyadenylation within the third intron and the production of a truncated non-functional transcript (Quesada et al., 2003). Recent studies estimated that 42–61% of multi-exon genes in *Arabidopsis* experienced AS process (Filichkin et al., 2010; Marquez et al., 2012), potentially affecting a wide range of cellular processes.

*Arabidopsis* flower development has been an excellent model system to understand the molecular control of plant development (Bowman et al., 1989; Coen and Meyerowitz, 1991; Smyth, 2005). In particular, gene activities and regulation during *Arabidopsis* flower development have been investigated with transcriptome, often using microarray; however, standard microarray chips are not designed to detect alternatively spliced transcripts. The rapid advances in next generation sequencing technologies have facilitated numerous studies on AS profiles in many species by RNA-Seq, such as *H. sapiens, D. melanogaster, S. cerevisiae, C. elegans, A. thaliana*, and *O. sativa* (Nagalakshmi et al., 2008; Filichkin et al., 2010; Graveley et al., 2010; Zhang et al., 2010; Ramani et al., 2011; Loraine et al., 2013). However, in contrast to the studies of AS in animals, analysis of AS in the context of plant development is still at an early stage.

To characterize the complexity of AS in *Arabidopsis* flower development, we have used RNA-Seq to analyse genome-wide AS events in *Arabidopsis* at three different stages of flower development. This is the first time to perform such analyses with ultra-high throughput technology, providing an accurate and comprehensive evaluation of AS profile for *Arabidopsis* flower development. Using several AS prediction approaches and manual verification, we estimated that approximately 25% multi-exon genes underwent AS, less frequent than the rate for *Arabidopsis* multiple tissues (Filichkin et al., 2010; Marquez et al., 2012). Among the events we found, intron retention (IR) (approximately 50%) was the most common type of AS compared to other types. The analysis further revealed that thousands of alternatively spliced transcripts were differentially expressed between different floral stages. We also identified many novel expressed regions considered unknown by the current annotations. Thus, our RNA-Seq data provide resources for comprehensive characterization of AS and gene expression in *Arabidopsis* flower development.

## Materials and methods

### Plant material collection, RNA isolation, and sequencing

Plant materials representing three stages of flower development were collected from *Arabidopsis thaliana* [inflorescent meristem (IM), flower development stages from 1 to 9, and flower development stage 12]. Considering the tiny structure of *Arabidopsis* IM and the difficulty it brings about to the sample collection, we used the IM from the *ap1cal* mutant as a substitution, which proliferates inflorescence meristems and results in a cauliflower appearance (Bowman et al., 1993). Total RNA was isolated for each of the three stages and treated with TRIzol (Invitrogen) according to the manufacturer's instructions, then was subjected to 50 bp single-end sequencing on a SOLiD 3 platform (http://www.appliedbiosystems.com). All SOLiD short reads have been submitted to NCBI Short Read Archive under accession number SRP035230.

### Read mapping and transcripts assembly

A total of 420 million 50 bp single-end reads were yielded for 5 samples from 3 stages. Reads from each sample were aligned to TAIR version 10 reference genome (www.arabidopsis.org) using Bowtie v0.12.7 (Langmead et al., 2009) and TopHat v1.3.2 (Trapnell et al., 2009) with a parameter “-C” for color space signal processing by SOLiD 3 platform. Intron sizes of predicted genes were limited to 50 and 5000 bp, to be consistent with those of annotated genes in the *Arabidopsis* genome (TAIR10), while the rest parameters were set as default. Cufflinks v0.93 (Trapnell et al., 2010) were then used to assemble the aligned reads, and to estimate the expression value of each assembled transcript.

### Alternative splicing events identification and classification

To identify, in each stage, the AS events and their corresponding types, the ASTALAVISTA algorithm (Foissac and Sammeth, 2007) was used to predict AS events and determine the types of the events from the GTF files output by Cufflinks. The parameters of minimum and maximum intron length were fixed at 50 and 5000 bp and the minimum reads covering novel identified transcripts should be no less than 15. Four AS types, namely intron retention (IR), exon skip (ES), alternative acceptor (AA), and alternative donor (AD), were extracted from the output of ASTALAVISTA for further analyses.

### Gene ontology enrichment analysis

To explore the functions of specifically expressed genes, we carried out GO enrichment analysis using the online AgriGO with Fisher's exact test (http://bioinfo.cau.edu.cn/agriGO/analysis.php). False discovery rate (FDR) correction was adopted with a threshold of 0.05 to reduce false positive prediction of enriched GO terms. A heatmap analysis for differentially expressed isoforms was implemented by using pheatmap (Pretty Heatmaps) function in an R package (pheatmap, version 0.74).

### Pfam domain annotation

The protein domain annotation was downloaded from the Pfam database (http://pfam.sanger.ac.uk) (Punta et al., 2012). For known genes/transcripts, we obtained the Pfam annotation from TAIR10 (www.arabidopsis.org), and other novel predicted transcripts were searched against Pfam-A database with *e*-value cutoff as 1e-5.

### Real-time PCR validation of gene expression and alternative splicing events

To verify the novel transcribed regions, we randomly selected 18 such regions to perform RT-PCR. *ACT2* gene (*AT3G18780*) was used as a positive control, for which primers were designed on two neighboring exons. Total RNAs of Col-0 inflorescences were obtained by ZYMO RESEARCH ZR Plant RNA Miniprep™, and reverse transcription was carried out by TAKARA PrimeScript™ RT Master Mix. Forty cycles were run on ABI Veriti® 96-well Thermal Cycler with Tiangen Taq DNA Polymerase (ET101) and proper primers. The same cDNA library was also used for real-time PCR validation of genes with different expression values. The real-time PCR was performed with TAKARA SYBR® Premix *Ex Taq*™ II (Tli RNaseH Plus) on ABI StepOnePlus™ Real Time system. Primers for these experiments are listed in Supplementary Table 7.

## Results

### Transcriptome profiling at three flower development stages of *A. thaliana*

**Figure 1:**
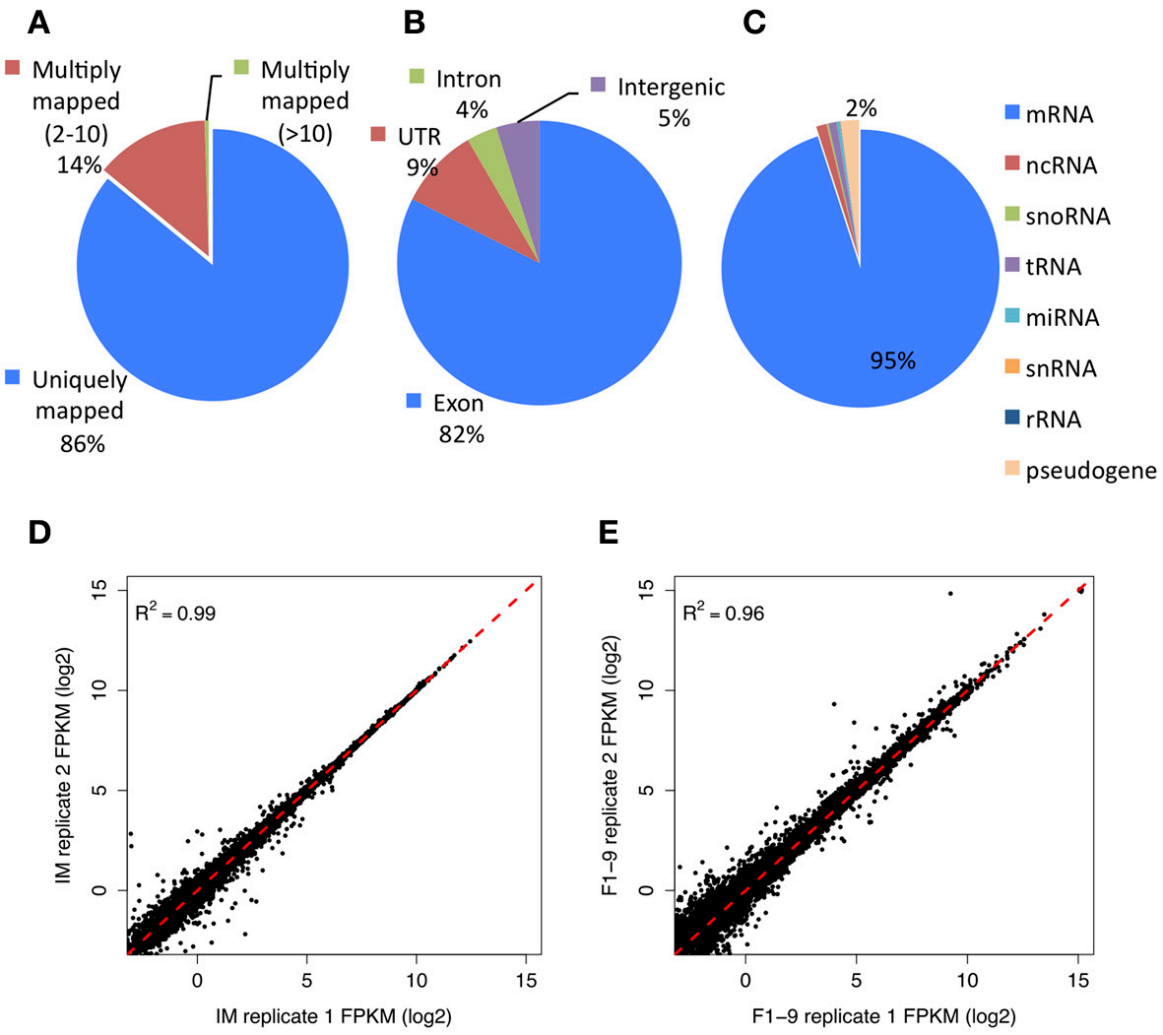
**The details of reads mapping and reproducibility of RNA-Seq data. (A)** Distribution of aligned reads, which are mapped either uniquely or to multiple positions on the genome. **(B)** Percentage of reads distributed to exons, introns, and untranslated or intergenic regions. **(C)** Classification of detected genes by annotated genomic features (percentage: mRNA 0.95; ncRNA 0.01; snoRNA 0.002; tRNA 0.009; miRNA 0.003; snRNA 0.0004; rRNA 0.0005; pesudogene 0.021). **(D)** Gene expression comparison between the two technical replicates for the inflorescent meristem (IM) stage. **(E)** Same comparison for flower development stage 1 to stage 9 (F1–9).

To investigate the transcriptomes of multiple stages in flower development, we isolated the total RNAs from three periods of flower development: IM stage, stage 1–9 (F1–9; early floral buds up to the time of meiosis) and stage 12 (F12; floral buds just before opening). Libraries were then constructed, and sequencing was performed on a SOLiD 3 platform, with one additional technique replicate for stages IM and F1–9. A total of 420 million high quality reads (50 bp, single-end) were obtained for the three stages of flower development, and 86% of them were mapped uniquely to the *Arabidopsis* reference genome (TAIR10) (Figure 1A). In addition, 82% of the mapped reads were aligned to protein-coding exonic regions, 9% to untranslated regions (UTR), 5% to intergenic regions and 4% to intron regions (Figure 1B). According to the annotation information from TAIR10, expression of 80% of the annotated genes was detected in our samples; 95% of the expressed genes were protein-coding while the rest included non-coding genes, micro RNA, transfer RNA, small nuclear RNA, and ribosome RNA (Figure 1C).

### The expression estimation of genes/transcripts across three flower development stages

The expression levels of genes and transcripts were estimated by applying Cufflinks (Trapnell et al., 2010) to short read alignment results for all three developmental stages. The comparison of the replicates of IM (*R*^2^ = 0.99) and F1–9 (*R*^2^ = 0.96) stages showed that the estimated levels of gene expression were highly consistent between the two replicates, especially for highly abundant genes (Figures 1D,E). The estimated gene expression was then used for further comparison among different flower development stages. The detailed statistics of read mapping were given in Supplementary Tables 1, 2. Number of assembled transcripts and their corresponding genes were shown in Table 1. The majority of the aligned reads were further assembled by Cufflinks, resulting in 22,827 fully assembled transcripts (of the 25,245 annotated genes). FPKM (Fragments Per Kilobase of transcript per Million mapped reads) was then calculated to estimate the expression value for each gene and/or transcript separately.

**Table 1:** **Number of assembled genes and transcripts of the three flower development stages**.

**Stages**	**Gene number**	**Transcript number**
IM	21,181	30,608
F1–9	22,137	29,759
F12	22,827	33,791

**Figure 2:**
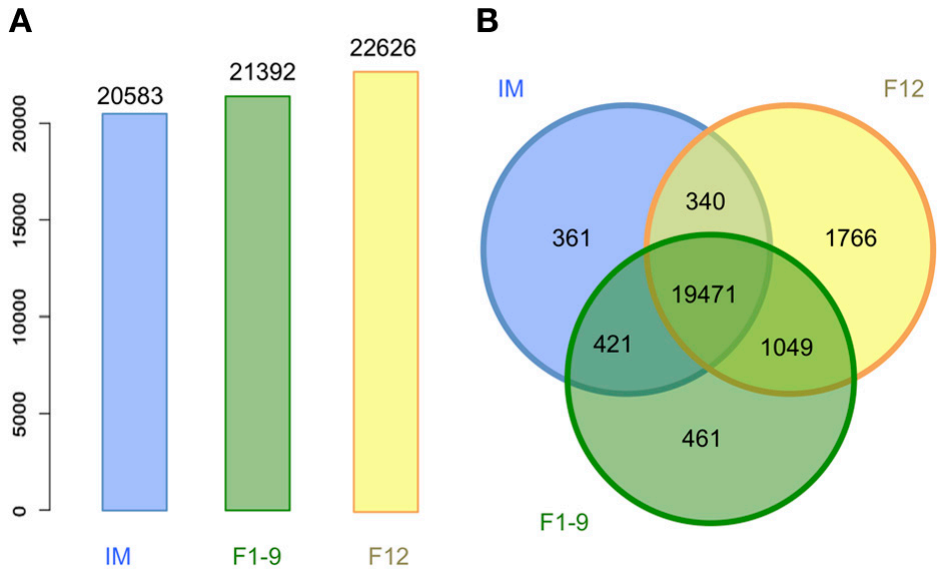
**Genes predicted based on RNA-Seq analysis for three floral developmental stages. (A)** Number of highly confident genes expressed in each stage. **(B)** Unique and shared genes among three developmental stages. IM represents inflorescent meristem, F1–9 for flower development stage 1–9, F12 for flower development stage 12.

To obtain an overview of the transcriptomes of flower development, we examined the distribution of gene expression values for each developmental stage. Each stage contained a group of genes with very low FPKM values, representing low expression or background. Taking a conservative approach, we used a cut-off of FPKM as 0.18 as a minimal expression value to avoid false positive estimation of gene expression, producing a unimodal distribution of genes expressed in each stage (Supplementary Figure 1). We detected 22,626 and 21,392 genes expressed at F12 and F1–9 stages, respectively (Figure 2A), much higher than the 14,833 and 14,460 genes previously reported for the same stages using microarray (Zhang et al., 2005). Compared with microarray technology, high throughput RNA-Seq technology could uncover the expression of many more genes.

We detected the expression of 20,583 genes at the IM stage, with a combined total of 23,860 genes (32,037 transcripts) expressed at the three stages of flower development; approximately 80% of these were detected at all three stages. We performed real-time PCR to validate the expression of 10 randomly selected genes, and the results were highly consistent with the estimates using RNA-Seq data (Supplementary Figure 2). In addition, 1049 genes were expressed at both the F1–9 and F12 stages but not in IM (Figure 2B), suggesting they are functionally important at relatively late stages of flower development. On the other hand, compared with the number of genes expressed only at IM (361) or at F1–9 (461), F12 had many more stage specific genes (1766), consistent with the fact that F12 contains more differentiated tissues and cells than the other two stages, particularly in the late stage of reproductive development, such as pollen and ovary development and so on.

### Identification of alternative splicing events and genes

To investigate the patterns of AS in flower development stages, we identified sequence reads supporting various types of AS events in our RNA-Seq data by using the ASTALAVISTA software (Foissac and Sammeth, 2007), which could detect the variations in the splicing structure and identify AS events by assigning each one an AS code, and has been applied to study AS in multiple *Arabidopsis* tissues (Marquez et al., 2012). In this study, we focused on four types of AS events: IR, AD, AA, and ES, by comparing RNA-Seq reads with annotated gene models. For example, we identified 4156 genes exhibiting AS among the 17,131 multi-exon genes of stage IM (Table 2). In total, we identified about 25.6% of TAIR10 annotated genes undergoing AS. Among the detected AS events, IR was the most abundant type (54.8 ± 0.98%), followed by AA (26.9 ± 0.2%), AD (13.1 ± 0.5%), and ES (5.2 ± 0.03%) (Table 3). These results were consistent with the recent findings in other studies (Filichkin et al., 2010; Zhang et al., 2010; Marquez et al., 2012). The observation that the percentage of IR events increased by approximately 14% from that in a recent genome-wide study in *Arabidopsis* using RNA-Seq (approximately 40% IR events) suggested that IR was probably important for floral development.

**Table 2:** **Estimation of alternatively spliced genes of the three stages**.

	**Flower stages (our study)**			**TAIR10**
	**IM**	**F1–9**	**F12**	
Multi-exon genes	17,131	18,048	18,326	22,352
AS genes	4156	3392	4466	5712
% of AS	24.3%	18.8%	24.4%	25.6%

**Table 3:** **Summary of the four types of alternative splicing events of the three development stages**.

	**IM**		**F1–9**		**F12**	
	**Events**	**Genes**	**Events**	**Genes**	**Events**	**Genes**
IR	2958 (54.8%)	2548 (52.9%)	2155 (55.0%)	1920 (50.4%)	3308 (56.6%)	2798 (54.7%)
ES	284 (5.2%)	274 (5.7%)	243 (5.3%)	235 (6.1%)	272 (4.7%)	265 (5.2%)
AA	1467 (26.9%)	1319 (27.5%)	1236 (27.1%)	1118 (29.3%)	1558 (26.7%)	1388 (27.1%)
AD	710 (13.1%)	667 (13.9%)	573 (12.6%)	540 (14.2%)	707 (12.1%)	667 (13.0%)

IR, Intron retention; ES, Exon skiping; AA, Alternative acceptor; AD, Alternative donor.

### Differential expression of isoforms across floral stages

**Figure 3:**
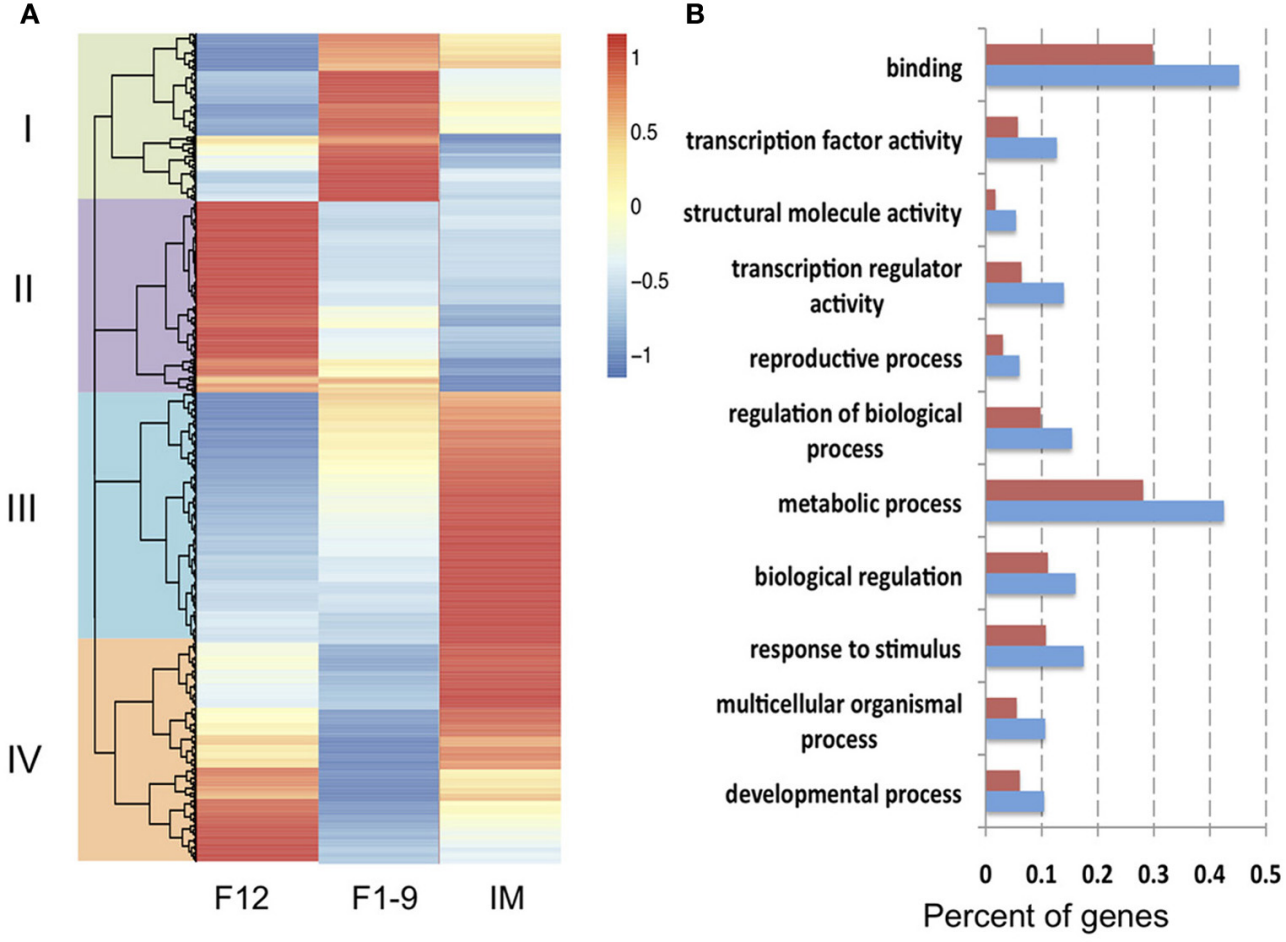
**Hierarchical clustering and function enrichment analysis of 1716 differentially expressed isoforms among three stages. (A)** Differentially expressed isoforms were clustered into four clusters: 359 isoforms in cluster I (with expression values as F1–9 > IM > F12), 460 isoforms in cluster II (F12 > F1–9 > IM), 401 isoforms in cluster III (IM > F1–9 > F12), and 526 isoforms in cluster IV (the others). Genes expressed higher than average are colored in red while lower in blue. **(B)** A list of enriched GO terms for genes in Cluster III. Red bars represent ratio of total annotated genes for each function, blue bars for differentially expressed genes within cluster III.

To investigate the possible role of alternatively spliced transcripts on flower development, expression levels of different isoforms of each gene were compared among the three stages. We found that 1716 alternatively spliced isoforms were differentially expressed between the three stages. These genes were further divided into four groups by hierarchical clustering algorithm by considering all the three stages simultaneously (Figure 3). Five hundred twenty-six isoforms (Cluster III in Figure 3) showed significantly higher expression levels at the IM stage than that at F1–9 and F12 stages, suggesting that these isoforms play roles in floral initiation. GO enrichment analyses were performed on this collection of isoforms and many functional categories were enriched including biological regulation, developmental processes, regulation of biological processes, and many biosynthetic processes. Among them, 19 genes were known to be important for flower development as demonstrated by genetic studies, such as *CIB5, SFH3, AGL71*, and *MAF3* (Mo et al., 2007; Liu et al., 2008; Dorca-Fornell et al., 2011; Rosloski et al., 2013) (Supplementary Table 5). Furthermore, many genes encoding transcription factors were expressed at higher levels in IM than in the other two stages, including members of bHLH, MADS, ERF/AP2, NAC, bZIP, B-box, and other families, strongly suggesting that the alternatively spliced variants of transcription factors significantly increase the complexity of transcription regulation networks during flower development.

From four clusters of differentially expressed splicing isoforms, we also found that genes involved in response to stress/stimulus were preferentially expressed, with 48 isoforms in cluster I, and 57, 55, 74 isoforms in cluster II, III, IV, respectively. Taken together, those differentially expressed isoforms were likely important for transcription regulation and for response to internal or external stimulus during flower development.

### Stage-specific splicing isoforms

Comparing to the differentially expressed isoforms, stage-specific AS isoforms might be more important (Lv et al., 2013). Before identifying alternatively spliced isoforms expressed specifically to each stage, we needed to find those genes exclusively expressed in each stage. The results showed that 13 genes out of 361 uniquely expressed genes were specifically alternatively spliced at stage IM, 13 out of 461 genes and 66 out of 1766 genes were alternatively spliced at stage F1–9 and F12, respectively.

**Figure 4:**
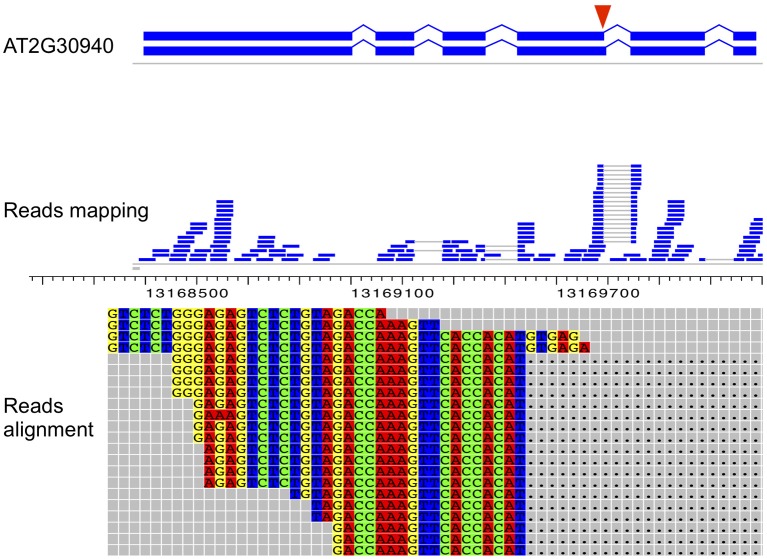
**An example of alternative spliced isoforms (of *AT2G30940*) expressed specifically at F12 stage.** The fourth exons of the two isoforms end at different sites (pointed out in a red triangle) according to gene annotations in TAIR10, and this alternative donor event was further confirmed by PCR validation on flower tissues. Each bar (middle panel) represents a short read mapped to the reference genome. Two ends of a split read, indicting a cleavage of an intron, are connected by a gray line. One of the two transcripts, *AT2G30940.1*, is only expressed in F12, revealed both by alignment details of reads mapped to an exon-exon junction and by RT-PCR validation (relative ratio approximately 220.5, consistent with that of FPKM).

Among the 66 genes with specific splicing isoform(s) at F12 stage, *AT2G30940*, encoding a protein kinase, has two isoforms due to an AD event. As shown in Figure 4, the presence/absence of six nucleotides at the end of the fourth exon was observed in different isoforms, which were distinguished by 18 reads mapped to the exon-exon junction. Compared with the first transcript (*AT2G30940.1*), the second transcript (*AT2G30940.2*) encodes a protein with 2 extra amino acids. In addition, both transcripts were expressed at stage F12 only, with *AT2G30940.1* more highly expressed than *AT2G30940.2* (FPKM values: 1.9 vs. 0.01), suggesting that they provide different levels of function for *Arabidopsis* flower development. Similar phenomenon was observed for another gene (*AT5G11400*) encoding kinase and a gene (*AT4G16162*) coding for a Leucine-rich repeat (LRR) protein. Interestingly, many genes coding for protein kinases were found to be involved in the regulation of constitutive and AS in plants and animals through phosphorylation and interaction with serine/arginine-rich proteins (Birney et al., 2007), the regulation of several kinase genes by usage of alternative promoters and/or AS has been studied in animals (Duncan et al., 1995, 1997). Still, AS events of kinase genes and their effects on flower development of plants are little known, thus calling for further studies (Marquez et al., 2012).

Another important gene *WRKY55* (*AT2G40740*) also expressed a splicing isoform specifically at stage F12. It is a member of the WRKY family, one of the largest families of plant-specific transcriptional regulators important for multiple plant processes. *WRKY55* can bind specifically to the W box (5′-(T)TGAC[CT]-3′), a *cis*-acting element mediating elicitor responses. Previous studies proved that *cis*-acting elements were responsible for AS of many genes (Black, 2003; Higashide et al., 2004; Stamm et al., 2005), e.g., *presenilin 2* (*PS2*) gene, which was abnormally alternatively spliced in a sample related to Alzheimer's disease (Higashide et al., 2004). For *WRKY55*, the ES event resulted in truncation of the WRKY domain, but the levels of expression were similar between these two isoforms (0.23 vs. 0.24; FPKM). Moreover, 36 other unknown functional genes with splicing isoforms specifically expressed at F12, providing particular materials for further genetic and genomic studies.

### Identification of novel transcribed regions from RNA-seq data

**Figure 5:**
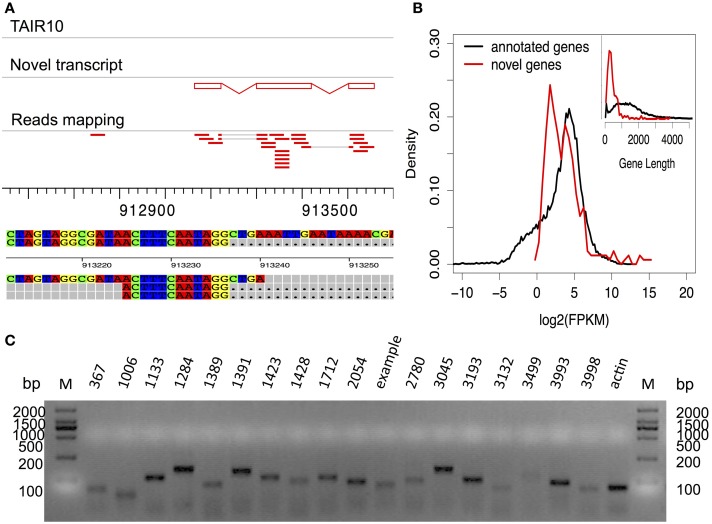
**Analysis of novel identified transcripts. (A)** Display of a novel transcribed region identified by RNA-Seq analysis. It includes three exons, supported by short reads (in red bars). Detailed alignment information (first exon-intron junction) is given in the lower panel. **(B)** Comparison of the expression values of novel transcripts and annotated genes. Comparison of gene lengths is given in the upper right panel. **(C)** RT-PCR validation of 18 randomly selected novel transcribed regions.

Compared with traditional technologies, RNA-Seq is able to identify novel transcribed regions without annotation from reads mapped to “intergenic” sequences. Considering the complex nature of *Arabidopsis* transcriptome, which contains many repetitive sequences from retro-transposons or recently duplicated genes, re-sequencing short reads from these variants could be wrongly mapped to non-allelic positions, making those regions marked as transcribed artificially. Thus only reads with mapping quality scores ≥20 were considered uniquely mapped and were used in these analyses. We discovered 337 regions with average length of 457 bp, which were shorter than that of known genes (2126 bp). An example of such putative novel genes with multiple exons is shown in Figure 5A. It is located on chromosome 1 between 913147 and 913739 with FPKM as 3.3 and was later verified by RT-PCR (Figure 5C). Each of the three exons of the gene was supported by five or more reads, while the two exon-exon junctions were also highly supported by multiple split-reads. We found that the expression distribution of these newly identified novel genes showed no significant difference from that of annotated genes (*p*-value >0.01; Figure 5B), suggesting that they are truly transcribed regions with high confidence. However, a majority of them (263 of 337) had only a single exon and were approximately 500 bp in length, with only a few longer than 1000 bp (Figure 5B). To verify those novel transcribed regions, we randomly selected 18 candidates for RT-PCR and 16 of them were confirmed, suggesting that the prediction of novel transcribed regions was highly accurate (Figure 5C). Furthermore, these novel identified genes lacked alternative splice variants. To determine whether they are functional will require further studies.

## Discussion

Previous studies used ESTs and microarrays to detect AS events, but these approaches are limited on both throughputs and sensitivities (Johnson et al., 2003; Iida et al., 2004). Recently developed RNA-Seq technologies with high throughput and reduced cost have greatly facilitated the comprehensive survey of gene expression and identification of AS events. In this study, we explored the transcriptomes of three different stages of *Arabidopsis* flower development using RNA-Seq, and detected the expression of approximately 24,000 genes from the three stages. The reproducibility of our transcriptome data was supported by two technical replicates of the IM and F1–9 stages. Expression of many more genes was detected in this analysis than in previous studies using microarray at the same stages of flower development, indicating RNA-Seq is more sensitive (Zhang et al., 2005, 2010).

Furthermore, the ability to assemble short reads into transcripts allowed us to examine previously annotated AS events and identify novel alternatively spliced events. From the analysis of three developmental stages, we predicted approximately 25% of multi-exon genes undergo AS. IR was the most abundant type of AS in our analysis, accounting for more than 50% of total AS events, slightly more than that found in other tissues of *Arabidopsis* or in other plants (Zhang et al., 2010; Marquez et al., 2012). Our results are consistent with those in the recent studies, on the percentage of AS types, on *Arabidopsis* using RNA-Seq (Marquez et al., 2012). The greater frequency of AS from other recent works in *Arabidopsis* is probably due to the fact that they estimated the AS genes using multiple tissues and/or stages under different growth conditions (Barbazuk et al., 2008; Marquez et al., 2012). To exclude the influence of sequencing technology, we compared the AS events identified from our SOLiD sequencing with those from unpublished Illumina paired-end data in our laboratory. We found AS events based on SOLiD sequencing (5845) were slightly fewer than those on Illumina platform (6132) and most of the predicted AS events were supported by both sequencing results, indicating these predictions were sequencing platform independent. The frequency of AS in plants are much lower than the estimates that up to 90% of human genes undergoing AS (Wang et al., 2008), suggesting that AS plays a less important role in gene regulation of plants than it does in animals, perhaps in part because plant genes can duplicate via several mechanisms, including whole-genome duplications (Blanc and Wolfe, 2004; Jiao et al., 2011).

Nevertheless, the AS isoforms identified here might be important for floral development since many of them are stage-regulated. We identified 1716 alternatively spliced isoforms, including some for genes encoding transcription factor, were differentially expressed among the three stages. Therefore, these genes coding for transcription factor are regulated by both AS and differential expression, providing additional layers of mechanisms for regulating flower development. Other processes affected by differentially expressed AS isoforms include response to stress or stimulus, consistent with previous reports (Dinesh-Kumar and Baker, 2000; Staiger and Brown, 2013). We also detected AS of several protein kinase genes, as also found previously (Duncan et al., 1995, 1997); furthermore, AS was reported to be regulated by phosphorylation of SR proteins (Lareau et al., 2007), indicating a complex mutual regulatory interaction between AS and protein phosphorylation. Whether AS during flower development is also regulated by protein phosphorylation is an interesting question awaiting further studies. We also found several stage-specific spliced genes, including *AT2G30940* that encoded a putative tyrosine kinase and produced AS transcripts by AD. The AS isoforms are similar in domain structure but have different expression levels. We found that *AT2G30940* was only expressed at stage F12 comparing IM and F1–9 stage, suggesting that it is important for late flower development.

Furthermore, AS may alter the structure of translated proteins, such as retention/deletion and elongation/reduction of protein domains, thereby affecting function. According to the annotation of Pfam database (Punta et al., 2012), we found that 19.2% of genes with annotated domains have length changes due to AS. Among these, genes encoding protein kinases were the most frequent, similar to previous observation (Punta et al., 2012); they have variable sizes from 61 to 377 aa that are associated with AS isoforms coding for proteins with truncated domains. Other domains with a wide range of sizes, e.g., K-box domain (from 42 to 100 aa), were also frequently affected by AS events (Supplementary Table 8).

Meanwhile, approximately 2.5% genes were identified to have their domains completely retained/deleted due to AS (see Supplementary Table 9 for the 10 most frequent types). For example, the WD40 domain defines a large family and is involved in several important biological processes including signal transduction, transcription regulation, and cell cycle control. We found that the WD40 domain is very frequently affected by AS, with various copy numbers of WD40 domains in different isoforms. Consistently, recent studies suggested that copy number changes of WD40 due to AS were found in many genes engaged in flowering transition (Ai et al., 2012) and other important regulatory network (Baurle and Dean, 2006). In addition, Zhou and his colleagues found that the WD40 domain of the *Arabidopsis* COP1 protein, which negatively regulates light-dependent development in the dark, could be deleted due to AS in a *cop1* mutant (Zhou et al., 1998). This alternatively spliced isoform always occurs in mature seeds and in germinating seedlings and light independent (Zhou et al., 1998). Our findings that the number and size of domains are affected by AS suggest that different consequences of AS are important for the regulation of gene function and might have experienced selective pressure during evolution.

Due to the nature of computational algorithms for *de novo* gene prediction, only typical gene models could be well-annotated, while sequences generated by RNA-Seq provide an alternative way to annotate genes, facilitating the correction of exon-intron boundaries and *de novo* gene detection (Wang et al., 2009). In a recent study of the fruit fly *Drosophila pseudoobscura*, most of the 669 new genes uncovered by RNA-Seq data are located on unassembled contigs, indicating that the annotation of *D. pseudoobscura* was incomplete (Palmieri et al., 2012). While in plants, people began to utilize RNA-Seq results from multi-tissue to annotate genes (Li et al., 2011). Our RNA-Seq analysis revealed 337 transcribed regions that were previously unknown and served to supplement the current *Arabidopsis* annotation. Some of these newly identified transcripts do not seem to encode proteins, suggesting that the RNA products might be the active form. Long non-coding RNAs have been identified from a number of organisms and are thought to play roles in regulating differentiation and stress response (Amor et al., 2009). Our results suggest that these transcripts could also play a role in regulating flower development.

In summary, our RNA-Seq analysis produced a comprehensive profile of AS during *Arabidopsis* flower development and provided valuable resources for investigating the regulation of gene expression and also facilitated further genomic and genetic studies.

## Conflict of interest statement

The authors declare that the research was conducted in the absence of any commercial or financial relationships that could be construed as a potential conflict of interest.
