# Supplementary material for: Alternative splicing during Arabidopsis flower development results in constitutive and stage-regulated isoforms
Source: Front Genet. 2014 Feb 12;5:25. doi: 10.3389/fgene.2014.00025 (PMC3921568; doi:10.3389/fgene.2014.00025)
Supplement: Supplementary file 1 [file DataSheet1.DOCX]

# SUPPLEMENTARY INFORMATION

**Supplementary Table 1.** Read alignment statistics.

**Supplementary Table 2.** Summary of transcript types detected by RNA-Seq.

**Supplementary Table 8.** Top 10 Pfam domains that are frequently truncated by AS.

**Supplementary Table 9.** Top 10 Pfam domains that are addition or deletion by AS.

**Supplementary Figure 1.** The expression value (FPKM) distribution across three stages.

**Supplementary Figure 2.** Validation of the expression of ten randomly selected genes using real-time PCR.

**Supplementary Table 1.** Read alignment statistics.

|  | **IM rep1** | **IM rep2** | **F1-9 rep1** | **F1-9 rep2** | **F12** |
| --- | --- | --- | --- | --- | --- |
| **Total reads** | 105,851,088 | 97,195,039 | 105,207,119 | 105,951,438 | 112,756,632 |
| **Aligned reads** | 30,046,238 | 28,780,823 | 32,008,796 | 45,959,881 | 47,302,403 |
| **Unique reads** | 27,411,421  (91.23%) | 26,263,581  (91.25%) | 29,067,152  (90.81%) | 42,394,906  (92.24%) | 43,503,157  (91.97%) |

**Supplementary Table 2**. Summary of transcript types detected by RNA-Seq.

| **Transcript**  **Types** | **IM rep1** | | **IM rep2** | | **F1-9 rep1** | | **F1-9 rep2** | | **F12** | |
| --- | --- | --- | --- | --- | --- | --- | --- | --- | --- | --- |
|  | **Gene number** | **Read number** | **Gene number** | **Read number** | **Gene number** | **Read number** | **Gene number** | **Read number** | **Gene number** | **Read number** |
| **mRNA** | 22,069 | 40,349,488 | 21,428 | 28,230,346 | 22,490 | 31,209,282 | 23,060 | 44,072,271 | 24,215 | 45,896,264 |
| **ncRNA** | 287 | 100,525 | 268 | 66,901 | 277 | 80,227 | 293 | 233,151 | 312 | 263,174 |
| **snoRNA** | 60 | 4,976 | 58 | 3,073 | 56 | 11,376 | 58 | 17,232 | 61 | 8,097 |
| **tRNA** | 94 | 977 | 42 | 312 | 55 | 3,152 | 153 | 8,117 | 90 | 1,845 |
| **miRNA** | 60 | 2,476 | 54 | 1,597 | 49 | 1,100 | 54 | 1,664 | 69 | 3,080 |
| **snRNA** | 11 | 253 | 8 | 32 | 9 | 51 | 11 | 701 | 13 | 366 |
| **rRNA** | 15 | 43,989 | 14 | 15,902 | 15 | 152,213 | 15 | 465,972 | 15 | 89,374 |
| **pseudo** | 351 | 70,046 | 301 | 37,420 | 338 | 42,363 | 411 | 83,491 | 470 | 72,497 |
| **All** | 22,947 | 40,572,730 | 22,173 | 28,355,583 | 23,289 | 31,499,764 | 24,055 | 44,882,599 | 25,245 | 46,071,523 |

ncRNA, non-coding RNA; snoRNA, small nucleolar RNA; snRNA, small nuclear RNA; pseudo, pseudogene.

**Supplementary Table 8.** Top 10 Pfam domains that are frequently truncated by AS.

| **Domain** | **No. isoform** | **Min length** | **Max length** | **Description** |
| --- | --- | --- | --- | --- |
| Pkinase | 176 | 61 | 377 | Protein kinase domain |
| K-box | 86 | 42 | 100 | K-box region |
| GRP | 86 | 75 | 98 | Glycine rich protein family |
| UQ_con | 74 | 41 | 149 | Ubiquitin-conjugating enzyme |
| Peptidase_S10 | 74 | 44 | 468 | Serine carboxypeptidase |
| RRM_1 | 66 | 26 | 81 | RNA recognition motif |
| Glyco_hydro_1 | 62 | 59 | 482 | Glycosyl hydrolase family 1 |
| P450 | 58 | 50 | 487 | Cytochrome P450 |
| Sugar_tr | 48 | 60 | 491 | Sugar (and other) transporter |
| PP2C | 46 | 50 | 309 | Protein phosphatase 2C |

Note: length is measured by amino acids; No. isoform means the number of isoforms that encode the domain in different lengths

**Supplementary Table 9.** Top 10 Pfam domains that are addition or deletion by AS.

| **Domain** | **No. isoform** | **Description** |
| --- | --- | --- |
| WD40 | 44 | WD domain, G-beta repeat |
| RRM_1 | 40 | RNA recognition motif |
| ubiquitin | 36 | Ubiquitin family |
| efhand | 26 | Phosphoinositide-specific phospholipase C |
| LRR_1 | 26 | Leucine Rich Repeat |
| Sugar_tr | 22 | Sugar (and other) transporter |
| Pkinase | 16 | Protein kinase domain |
| PPR | 16 | Pentatricopepetide repeat domain |
| Myb_DNA-binding | 16 | Myb-like DNA-binding domain |
| LIM | 14 | LIM domain |

Note: No. isoform means The number of isoforms that encode the domain of different number

**Supplementary Figure 1.** The expression value (FPKM) distribution of three stages.

**Supplementary Figure 2.** Validation of the expression of ten randomly selected genes using real-time PCR.


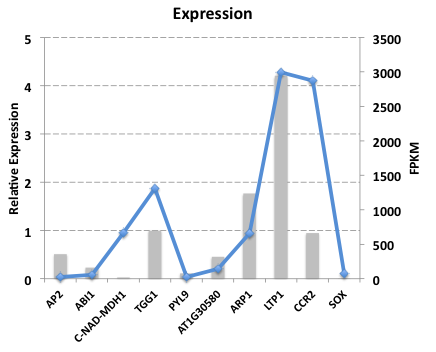


Grey bars represent relative expression ratios, while blue lines represent RNA-Seq expression estimation in FPKM.
